# SPH modelling of depth‐limited turbulent open channel flows over rough boundaries

**DOI:** 10.1002/fld.4248

**Published:** 2016-05-25

**Authors:** Ehsan Kazemi, Andrew Nichols, Simon Tait, Songdong Shao

**Affiliations:** ^1^ Department of Civil and Structural EngineeringThe University of SheffieldMappin StreetSheffieldS1 3JDUK

**Keywords:** SPH, turbulence, open channel flow, rough bed, drag force, inflow/outflow boundaries

## Abstract

A numerical model based on the smoothed particle hydrodynamics method is developed to simulate depth‐limited turbulent open channel flows over hydraulically rough beds. The 2D Lagrangian form of the Navier–Stokes equations is solved, in which a drag‐based formulation is used based on an effective roughness zone near the bed to account for the roughness effect of bed spheres and an improved sub‐particle‐scale model is applied to account for the effect of turbulence. The sub‐particle‐scale model is constructed based on the mixing‐length assumption rather than the standard Smagorinsky approach to compute the eddy‐viscosity. A robust in/out‐flow boundary technique is also proposed to achieve stable uniform flow conditions at the inlet and outlet boundaries where the flow characteristics are unknown. The model is applied to simulate uniform open channel flows over a rough bed composed of regular spheres and validated by experimental velocity data. To investigate the influence of the bed roughness on different flow conditions, data from 12 experimental tests with different bed slopes and uniform water depths are simulated, and a good agreement has been observed between the model and experimental results of the streamwise velocity and turbulent shear stress. This shows that both the roughness effect and flow turbulence should be addressed in order to simulate the correct mechanisms of turbulent flow over a rough bed boundary and that the presented smoothed particle hydrodynamics model accomplishes this successfully. © 2016 The Authors International Journal for Numerical Methods in Fluids Published by John Wiley & Sons Ltd

## 1 Introduction

Because all natural river flows around the world are turbulent and the channel beds are often composed of large‐scale, potentially mobile, rough elements such as sand and gravel particles, the study of turbulent open channel flows over rough beds is of significant engineering interest. This interest has motivated researchers to carry out various studies to explore the flow behaviour near the solid–fluid interface in laboratory experiments or to simulate the effect of bed roughness on the flow by numerical methods. The solution of fundamental hydrodynamic equations has become a popular numerical technique in modelling turbulent flows because it can provide time‐dependent details of the flow characteristics such as velocities, pressures and transport properties. In turbulence modelling of open channel flows, the Reynolds‐averaged Navier–Stokes (N‐S) equations or space‐filtered large eddy simulation (LES) equations have been widely used, where the large eddies are resolved and the small ones are modelled by an appropriate model, usually the eddy‐viscosity model. The eddy‐viscosity model relates the turbulent shear stress to the local strain rate through an eddy‐viscosity *ν_t_* based on the Boussinesq approximation. A simple, economical and practical approach to evaluate *ν_t_* is using a mixing‐length model that is known as the zero‐equation model. In this approach, the eddy‐viscosity is related to the mean strain rate from Prandtl's theory by using a turbulence characteristic length *l_m_* as follows:

(1) $$\nu_{t}={l_{m}}^{2}\left(\frac{dU}{dz}\right)\text{,}$$

where *U* is the mean streamwise velocity and *l_m_* is the mixing length. Although the mixing‐length model is easy to use, it lacks the universality and is not applicable to complicated flows (e.g. 3D non‐uniform flows with disturbed free surface) where the distribution of turbulence length scale *l_m_* is not known. A well‐known turbulence model that is commonly used for such complicated flows is the two‐equation *k–ε* model where a wall function technique is usually used to estimate the flow in the shear boundary layer. Although this model has the advantage of including the effect of flow history and transport on the turbulence, it meets difficulties in treating rough wall boundaries because the near‐bed logarithmic law does not hold anymore when large roughness elements exist. This has also been investigated by Nikora *et al.* 1, who showed that in the interfacial sub‐layer, which is the flow region between the roughness crest and trough, the velocity profile can be either constant, exponential or linear based on the flow conditions, relative submergence and roughness geometry. Another deficiency of the wall function approach has been addressed by Nicholas 2, in that the shear stress could not be accurately reproduced by a wall function approach because of the mesh resolution problems in the region near the rough bed. On the other hand, the LES modelling approach is based on the spatially averaged equations where usually a sub‐grid‐scale model is used to relate the turbulent eddy‐viscosity with the local flow strain rate by using the Smagorinsky model 3.

Different approaches have been adopted to account for the roughness effect in numerical modelling of turbulent flow over rough walls. Some have been developed based on modifying the turbulence model near the rough boundary; while in some others, separate models have been used, for example, the roughness effect being formulated on the basis of a drag force equation. Van Driest 4 proposed a modification to his mixing‐length formula originally derived for hydraulically smooth walls. Based on this modification, the shear stress was increased near the wall because of the existence of the roughness elements. Rotta 5 proposed a different modification to the van Driest formula by introducing a shift in the wall coordinate in order to increase the shear stress near the wall to take the roughness effect into account. Despite their simplicity, these models have provided a physical description of flow pattern near the wall and have been applied in several studies for calculation of boundary layers on rough walls, for example, in Cebeci and Chang 6. However, their modified model is not suitable in cases where the wall is composed of large‐scale discrete rough elements such as in gravel bed rivers. Hence, other researchers have explicitly modelled the roughness effect by using a drag‐based model in which a sink term of the form drag is added to the momentum equations to address the form drag effect on the near‐wall flow. Christoph and Pletcher 7 and Taylor *et al.* 8 used such models to simulate the roughness effect together with a mixing‐length model to account for the turbulence. Wiberg and Smith 9 divided the total shear stress into a fluid shear component and a form‐induced component and used a mixing‐length model for the former and a drag force equation for the latter to calculate the velocity distributions in a steep stream over coarse gravel beds. Besides these, Cui *et al.* 10, Carney *et al.* 11 and Zeng and Li 12 are some other examples of studies in which the drag concept has been applied to model the effect of wall roughness on the flow. Among them, Zeng and Li 12 used a wall function approach to treat the shear boundary for small‐scale rough bed elements and a drag force model for large‐scale rough beds when the wall function approach was unable to reproduce the correct velocity distributions.

Recently mesh‐free particle methods, for example, smoothed particle hydrodynamics (SPH), have been used in fluid flows because of their advantages in dealing with the large deformation of free surfaces and solid–fluid interfaces. SPH can model flows by tracking each individual particle without numerical diffusion and has been used in various applications such as wave breaking, fluid impact and flow‐structure interactions. As examples of hydraulic engineering‐related SPH studies, Gotoh *et al.* 13 and Shao and Gotoh 14, 15 should be mentioned. Recently, several other studies have been carried out focussing on the enhancement of the accuracy of particle methods in fluid flows, for example, Khayyer and Gotoh 16, Lind *et al.* 17 and Gotoh *et al.* 18. Besides, further studies have also been performed to improve the modelling of the effect of wall and free surface boundary conditions, for example, Ferrand *et al.* 19, Leroy *et al.* 20 and Tsuruta *et al.* 21.

However, very few cases have involved open channel flows, although some pioneering works in this field have been reported such as by Federico *et al.* 22 and Fu and Jin 23. Because of this, turbulence and rough bed issues have not been effectively solved for the type of flows found in shallow rivers with a rough boundary. For turbulence models in SPH, the earliest and most comprehensive work could be attributed to Gotoh *et al.* 24 and Violeau and Issa 25. The former proposed a novel eddy‐viscosity‐based sub‐particle‐scale (SPS) turbulence model for a turbulent jet based on the moving particle semi‐implicit (MPS) method, in which the turbulent quantities were validated but the model applications were mainly based on the smooth wall. The latter developed two Reynolds‐averaged N–S turbulence models and also applied a LES approach to simulate more complex turbulent free‐surface flows.

As for the shear boundary treatment, Violeau and Issa 25 used a wall function approach to impose the logarithmic velocity distributions near the wall. Besides, Lopez *et al.* 26 developed an SPH model with variable artificial viscosity to simulate hydraulic jumps, and they applied a Lennard–Jones repulsive force on the bed particles to produce a ‘numerical’ resistance on the near‐wall flow. Sahebari *et al.* 27 and Fu and Jin 23 used the SPS model with Smagorinsky constant *C_s_* = 0.15 in their MPS simulations of open channel flows. Sahebari *et al.* 27 did not treat the bed roughness effect, while Fu and Jin 23 adjusted the velocity of dummy particles near the bed boundary to take the roughness effect into account. In this way, different types of bed conditions, including smooth, intermediately rough and fully rough beds, have been studied. Chern and Syamsuri 28 also used the SPS turbulence modelling approach but with *C_s_* = 0.12 and simulated hydraulic jumps over corrugated beds by using SPH. They treated the wall boundaries of smooth, triangular, trapezoidal and sinusoidal shapes by using lines of the particles and applied a repulsive force similar to that of Lopez *et al.* 26. De Padova *et al.* 29 employed an eddy‐viscosity model based on the mixing‐length concept for flow turbulence to simulate hydraulic jumps in a large channel by SPH. Nevertheless, no bed boundary treatment was included in their model. Arai *et al.* 30 applied a wall function to estimate the near‐wall velocity in their MPS model with a Smagorinsky‐based eddy‐viscosity model for turbulence in a LES of turbulent channel flows. A more physically sound rough bed modelling approach was initiated by Gotoh and Sakai 31 for a breaking wave inside a porous medium. They pointed out that a drag force equation could be the most appropriate way to address the bed roughness. Khayyer and Gotoh 32, 33 developed a more mature drag force model to address the wall friction effect for a dam break flow over a wet bed. Besides, it is also worth mentioning that recently, quite a few influential works have been carried out in open channel flows by using the concept of shallow water SPH 34, 35, 36.

In two recent studies, Mayrhofer *et al.* 37, 38 effectively investigated the turbulence modelling of wall‐bounded flows using SPH. Mayrhofer *et al.* 38 introduced an additional volume diffusion term into the continuity equation in order to treat the noises that arise as a result of the SPH discretization. They used an eddy‐viscosity model with a mixing‐length approach to estimate the additional diffusion term. More recently, Mayrhofer *et al.* 37 applied the SPH method in a direct numerical simulation (DNS) as well as LES of 3D wall‐bounded turbulent channel flows and revealed interesting findings. They firstly performed a quasi‐DNS of a 3D channel flow based on SPH and achieved good agreement with the reference data except for some near‐wall oscillations. Then they carried out a LES of a channel flow with friction *Re* number (*Re_τ_*) of 1000 using SPH with the unified semi‐analytical wall boundary condition and an eddy‐viscosity model with the Smagorinsky constant *C_s_* = 0.065 for the unresolved part of the turbulence. In contrast to the DNS, the result of the LES was very poor. In order to investigate the insufficiency of their LES, they considered a Taylor–Green vortex case and stated that the failure was traced back to the SPH collocated discretization effect on the pressure–velocity interactions. Finally, they concluded the LES of a channel flow is still not possible with the present SPH formulation because of the problems inherent in the standard SPH discretizations.

In grid‐based LES, a variable resolution is usually adopted so as to use a much finer mesh near the wall boundary in order to resolve the near‐wall flow scales, while in SPH, a non‐variable homogenous discretization has to be used. Hence, a wall function is usually applied, such as in the studies of Violeau and Issa 25, Arai *et al.* 30 and Mayrhofer *et al.* 37, to account for the wall effect.

In a most recent study in this area, Kazemi *et al.* 39 completed a comprehensive review on the numerical modelling of turbulent open channel flows over rough bed boundaries. They focused on the procedures of turbulence modelling and rough bed boundary treatments and reviewed mesh‐free particle models that have been developed for these purposes. They remarked the deficiency of the eddy‐viscosity models with the Smagorinsky constant in treating the turbulence effect in SPH simulation of highly turbulent channel flows over rough boundaries, and also the insufficiency of the wall functions in treating the rough wall boundaries, which occurs because the near‐wall velocity profile is not always logarithmic when the boundary consists of large roughness elements. Accordingly, the SPH method was recommended to be coupled with a mixing‐length model for turbulence and a drag force equation model to treat the shear boundary near beds with large‐scale roughness. In the present study, the proposed model is further developed and used to investigate the effects of bed roughness in different regimes of turbulent flow over rough bed boundaries. In summary, we will use the fundamental eddy‐viscosity‐based SPS model proposed by Gotoh *et al.* 24 but adopt a mixing‐length approach to realistically calculate the eddy‐viscosity to improve the turbulence model performance in open channel flows. As for the drag force model, we will improve it by including a shape function in the drag force equation to account for the shape of bed roughness elements so as to more realistically evaluate the bed surface geometrical conditions. Also, an efficient inflow/outflow boundary treatment is used to generate an accurate and stable uniform flow along the channel. In model applications, the depth‐limited flows with different regimes but with the same bed roughness are simulated, and the velocity and shear stress profiles are validated by experimental data for 2D rough bed turbulent flow. Following Cheng *et al.* 40, we consider the depth‐limited condition as when the ratio of the bed roughness size to the water depth is significant. As far as we know, no documented SPH works have reported the quantification of such flow information for turbulent open channel flows over rough beds for conditions similar to those found in gravel bed rivers.

## 2 Numerical Modelling Scheme

### 2.1 Governing equations

The governing equations are the two‐dimensional continuity and momentum equations in the Lagrangian framework. An additional term to represent the form drag of the bed particles is included. This term as well as the turbulent shear term is not needed in a DNS. The final equation reads

(2) $$\frac{D\rho }{Dt}=-\rho \nabla \cdot u\text{,}$$

(3) $$\frac{Du}{Dt}=-\frac{1}{\rho }\nabla P+g+\nu_{0}\nabla^{2}u+\frac{1}{\rho }\nabla \cdot \tau_{t}+\frac{1}{\rho }\tau_{d}\text{,}$$

where *t* is the time, *ρ* is the fluid density, **u** is the velocity, *P* is the pressure, **g** is the gravitational acceleration, *ν*_0_ is the kinematic viscosity coefficient, **τ** *_t_* is the turbulence stress tensor and **τ** *_d_* is the form drag‐induced shear stress from the rough bed.

To model the turbulence stress, an SPS model based on the eddy‐viscosity assumption 24 is used as follows:

(4) $$\frac{\tau_{ij}}{\rho }=2\nu_{t}S_{ij}-\frac{2}{3}k\delta_{ij}\text{,}$$

where *i* and *j* denote the 2D coordinate components, *τ_ij_* is the component of shear stress tensor **τ** *_t_*, *S_ij_* is the component of strain tensor **S** calculated by Eq. (5), *ν_t_* is the turbulence eddy‐viscosity, *k* is the turbulence kinetic energy calculated by Eq. (6) and *δ_ij_* is the Kronecker delta function.

(5) $$S_{ij}=\frac{1}{2}\left(\frac{\partial u_{i}}{\partial x_{j}}+\frac{\partial u_{j}}{\partial x_{i}}\right)\text{,}$$

(6) $$k=\nu_{t}\left(\frac{\partial u_{i}}{\partial x_{i}}+\frac{\partial u_{j}}{\partial x_{j}}\right)\text{,}$$

where *x* and *u* are the position and velocity components, respectively. In SPH, the turbulence eddy‐viscosity *ν_t_* is usually estimated by the Smagorinsky model 3, following the initiatives of Gotoh *et al.* 24, as follows:

(7) $$\nu_{t}={\left(C_{s}\Delta \right)}^{2}\left|S\right|\text{,}$$

where *C_s_* is the Smagorinsky constant, usually taken to be between 0.1 and 0.15, Δ is the characteristic length scale of eddies (filter width), which is taken as the particle spacing, and $\left|S\right|=\sqrt{S:S^{T}}$ is the local strain rate. It should be noted that the turbulence has a three‐dimensional nature, and in particular for the spatially averaged LES‐based modelling and consideration of SPS turbulence closure, the three‐dimensional characteristics of turbulence should play an important role. However, in the present simulations of open channel uniform flow, the flow is dominated by the streamwise shear stress and vertical 2D momentum exchange, while the lateral influence is quite small so as to be reasonably neglected in this study.

Equation (7) has been used with SPH in several coastal hydrodynamic applications, and the accuracy has proved to be satisfactory. However, its applicability in open channel flows with SPH has been under‐reported. In our previous computational experience 39, the Smagorinsky‐based SPS model with *C_s_* = 0.15 was not able to reproduce the correct shear mechanism in a uniform open channel flow over a rough wall. Also, in the study of Mayrhofer *et al.* 37, using an eddy‐viscosity model with a Smagorinsky constant *C_s_* = 0.065 in the SPH‐LES showed very poor results with an overestimation in the streamwise velocity. They pointed out that the failure was related to the pressure–velocity interactions of vortices and concluded that this problem is inherent in the standard SPH discretization.

We also carried out some simulations with the Smagorinsky constant *C_s_* = 0.15 to investigate this issue. The results are presented in Section 3.4, which shows the failure of the SPH using the standard Smagorinsky eddy‐viscosity model for turbulence. The failure is attributed to the deficiency of the standard Smagorinsky model in dealing with the cases in which sharp changes take place in the flow velocity, like the one studied in present work. Further discussions on this issue will be provided in Section 3.4. An alternative approach adopted here is then to explore the concept of a standard mixing‐length model to estimate the turbulent eddy‐viscosity in present SPH scheme in order to recover the part of the turbulence that cannot be captured by the standard Smagorinsky model with a *C_s_* being around 0.15. Accordingly, the eddy‐viscosity is formulated as follows:

(8) $$\nu_{t}={l_{m}}^{2}\left|S\right|\text{,}$$

where the mixing length *l_m_* is calculated by the Nezu and Rodi 41 empirical formula as follows, which has been derived on the basis of physical measurements.

(9) $$\frac{l_{m}}{H}=k\sqrt{1-\xi }\;\left[\frac{1}{\xi }+\pi \;\Pi \;\sin\;\left(\pi \;\xi \right)\right]\text{,}$$

where *H* is the water depth, *k* is the von‐Karman constant and *ξ* = *z*/*H* is defined in which *z* is the vertical coordinate, and Π is the Coles parameter. Π has been introduced to describe the deviation from the log law in the outer region. This parameter comes from an empirical wake function added to the velocity log law by Coles 42. Coleman 43 has also expressed that the deviation in the outer layer from the log law should not be accounted for by adjusting the von‐Karman constant *k* and/or the integration constant (*B_r_* in Eq. (21)) but rather by adding a wake function to the log law equation (Eq. (21)). However, in the present study, a value of 0.41 is adopted for *k*, and Π is assumed to be 0 so that the following Eq. (10) is used to estimate the mixing length that is a simplified form of Eq. (9). This formula has also been used in the studies of Violeau and Issa 25 in modelling the turbulent open channel flows by the SPH method.

(10) $$l_{m}=kz\sqrt{1‐z/H}\text{.}$$

Considering *x* and *z* as the streamwise and vertical coordinates in a strongly 2D uniform open channel flow, and *u* and *w* as the streamwise and vertical velocity components, respectively, Eq. (8) would be equivalent to Prandtl's theory (Eq. (1)), as the local strain rate |**S**| is approximately equivalent to ∂*u*/∂*z* because of the other velocity gradients such as ∂*u*/∂*x*, ∂*w*/∂*x* and ∂*w*/∂*z* being significantly smaller.

To account for the effect of bottom roughness, the form drag‐induced shear term **τ** *_d_*/*ρ* should be added to the momentum equation (3), because the macroscopic N‐S equations are considered rather than a high spatial resolution (DNS) is solved for the refined flow details within the roughness region, which could use considerable CPU resources. **τ** *_d_* will be calculated by following Eq. (11), where **F** *_d_* is the drag force exerted on the fluid particle from the rough bed, which is assumed to be equal to and in the opposite direction of the force from the fluid particle acting on the bed. *A_τ_* is the bed‐parallel, planar area affected by the fluid particle. Furthermore, the drag force **F** *_d_* will be calculated by Eq. (12), where *C_d_* is the drag coefficient, *A_d_* is the planar cross‐sectional area and *W_d_* is a non‐dimensional shape function accounting for the geometry of the bed roughness. The quantifications of relevant drag parameters will be detailed in Section 2.3.

(11) $$\tau_{d}=\frac{F_{d}}{A_{\tau }}\text{,}$$

(12) $$F_{d}=-\frac{1}{2}C_{d}W_{d}\rho A_{d}u\left|u\right|\text{.}$$

### 2.2 Discretization of equations by smoothed particle hydrodynamics

The numerical scheme based on the weakly compressible SPH (WCSPH) method is used to discretize the governing equations. SPH is a Lagrangian particle method that was developed by Gingold and Monaghan 44 initially for astrophysical problems. Since then it has been widely used for simulating fluid flows. In the SPH approximation, any variable, for example *A*(**r**), can be estimated by the following integral interpolant equation as:

(13) $$A\left(r\right)=\int_{\Omega }A\left(r\prime \right)W\left(|r-r\prime |,h\right)dr\prime \text{,}$$

where Ω is the volume of the integral, **r** is the particle position, **r**′ denotes the particle coordinate, *h* is the smoothing length and *W*(|**r − r**′|,*h*) is the weighting or kernel function. The aforementioned equation can be expressed in the following discretized form to calculate *A*(**r**) at the position of particle *a*:

(14) $$A\left(r_{a}\right)=\sum_{b}m_{b}\frac{A\left(r_{b}\right)}{\rho_{b}}W\left(|r_{a}-r_{b}|,h\right)\text{,}$$

where *a* and *b* are the reference particle and its neighbour and *m_b_* and *ρ_b_* are the mass and density of neighbouring particle *b*, respectively. The derivative of *A*(**r**) in the *x_j_* direction can be approximated by the following:

(15) $$\frac{\partial A\left(r_{a}\right)}{\partial x_{j}}=\sum_{b}m_{b}\frac{A\left(r_{b}\right)}{\rho_{b}}\frac{\partial W\left(|r_{a}-r_{b}|,h\right)}{\partial x_{j}}\text{.}$$

By using the aforementioned SPH formulations, the governing equations (Eqs (2) and (3)) are discretized as follows for the computations of density and velocity of the particles:

(16) $$\frac{D\rho_{a}}{Dt}=\rho_{a}\sum_{b}\frac{m_{b}}{\rho_{b}}u_{\text{ab}}\cdot \nabla_{a}W_{\text{ab}}\text{,}$$

(17) $$\begin{array}{l} \frac{Du_{a}}{Dt}=-\sum_{b}m_{b}\left(\frac{P_{a}}{{\rho_{a}}^{2}}+\frac{P_{b}}{{\rho_{b}}^{2}}\right)\nabla_{a}W_{\text{ab}}+g+\sum_{b}m_{b}\frac{4\nu_{0}}{\left(\rho_{a}+\rho_{b}\right)}\frac{r_{\text{ab}}\cdot \nabla_{a}W_{\text{ab}}}{{\left|r_{\text{ab}}\right|}^{2}+\eta^{2}}u_{\text{ab}}\\ \;+\sum_{b}m_{b}\left(\frac{\tau_{a}}{{\rho_{a}}^{2}}+\frac{\tau_{b}}{{\rho_{b}}^{2}}\right)\cdot \nabla_{a}W_{\text{ab}}+\frac{1}{\rho_{a}}{\left(\tau_{d}\right)}_{a}\text{,} \end{array}$$

where **u** *_ab_* = **u** *_a_* **− u** *_b_* and **r** *_ab_* = **r** *_a_* − **r** *_b_* are defined, ∇*_a_W_ab_* is the gradient of the kernel function between particles *a* and *b* with respect to the position of particle *a* and *η* is a small number used to prevent singularity. In the present WCSPH model, the following Eq. (18) is used to link the continuity equation with the momentum equation to compute the fluid pressure from the change in particle density in an explicit way as follows:

(18) $$P={c_{0}}^{2}\left(\rho -\rho_{0}\right)\text{,}$$

where *ρ*_0_ is the reference density and *c*_0_ is the speed of sound. In a WCSPH numerical scheme, it is assumed that the flow is slightly compressible so the speed of sound should be chosen to be around 10 times of the bulk flow velocity to ensure the fluid compressibility being less than 1%. Finally, *ρ*_0_ and *c*_0_ are respectively taken as 1000 kg/m^3^ (water density) and 16 m/s as a common practice in the computations. Since the WCSPH is known to result in considerable numerical noise in the pressure field, a special treatment (density filtering, delta‐SPH terms, etc.) could be undertaken to improve the performance. Therefore, the present WCSPH simulations have been performed using a Shepard density filter to minimize the pressure noise at every 30 computational time steps. The solution method using a predictor–corrector scheme 45 is implemented to solve the governing equations and update the density, velocity and position of the particles. The selection of the computational time step follows the Courant–Friedrichs–Lewy condition.

### 2.3 Boundary conditions

**Figure 1:**
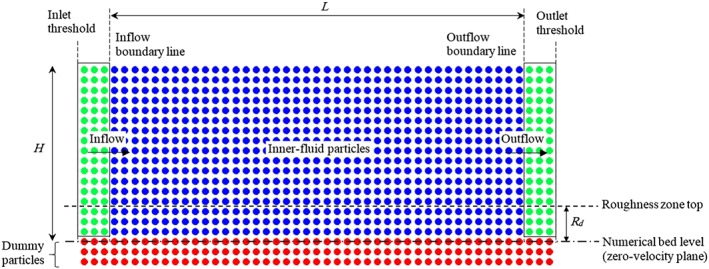
A schematic view of the computational domain and boundary conditions.

The computational domain boundaries including the free surface, rough boundary and inflow/outflow boundaries are shown in Figure 1. There is no special treatment for the free surface boundary in the SPH method because the particles are automatically tracked.

#### 2.3.1 Treatment of inflow/outflow boundary

Recently, some pioneering works have been carried out on the treatment of inflow/outflow boundary conditions in SPH, for example, Federico *et al.* 22, Aristodemo *et al.* 46 and Tan *et al.* 47. In present study, a similar technique has been adopted but with the difference in that the inflow particle velocities are linked with those of the inner fluid particles, so that the flows are evolved naturally without any prescription of the inflow velocity. For the inflow and outflow boundaries, several layers of particles are located beyond the boundary line but within the threshold line to cover the truncated kernel area of the inner‐fluid particles near the boundary (Figure 1). The governing equations are not solved for these particles, but they move according to the flow conditions inside the inner‐fluid domain. In this way, the velocity and pressure of inflow/outflow particles are evolved through calculations rather than being allocated the prescribed values. The proposed technique is suitable for cases where the inflow and/or outflow conditions are not known and need to be determined through the simulations. One example is the gravity‐driven flow over a sloping channel bed that is considered in the present study. To generate an open channel uniform flow, the appropriate flow conditions need to be achieved at the inflow boundary, that is, the gradients of the velocity and pressure in the streamwise direction *x* should be 0 at the boundary line, represented by the following:

(19) $$\begin{array}{c} \frac{\partial u}{\partial x}=0,\\ \frac{\partial P}{\partial x}=0\text{.} \end{array}$$

**Figure 2:**
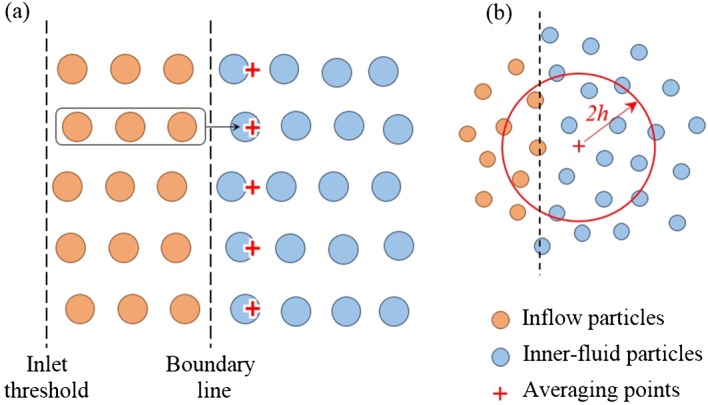
(a, b) Inflow boundary treatment.

To satisfy these conditions in an SPH computation, the properties of the inflow particles (e.g. velocity and density) are set equal to those of the inner‐fluid particles near the inflow boundary line. To do so, an averaging point is first defined for each inflow particle at the same elevation but inside the inner‐fluid region, with a distance of *d_p_*/2 from the boundary line as shown in Figure 2(a), where *d_p_* is the SPH particle size. Then the velocity and density of the inner‐fluid particles are averaged over a kernel area onto these points and set as the velocity and density of the corresponding inflow particles (Figure 2(b)). Therefore, the gradient of velocity as well as the density is 0 at the boundary. Because the pressure is calculated by using Eq. (18), the zero pressure gradient is also satisfied, and thus, the flow uniformity is achieved. When an inflow particle crosses the boundary line and enters the inner‐fluid region, it becomes an inner‐fluid particle, and the governing equations are solved for it in the next time step. Meanwhile, an additional inflow particle is generated with the same properties at the inlet threshold line for the same elevation (Figure 2(a)). In this way, the inflow region bounded by the inlet threshold line and the inner‐fluid area acts like a particle generator to reach a uniform flow condition at the boundary. For consistency, the same kernel function and smoothing length of the inner‐fluid SPH calculations are used for the averaging process in Figure 2(b). The novelty of the proposed inflow boundary treatment over that of existing approaches is that the flow is naturally evolved through the numerical simulations without being given a prescribed inflow velocity, so the model can be applied to a much wider range of hydraulic applications in which the inflow information is unknown.

At the outflow boundary, the uniform flow condition should also be satisfied to keep the uniformity of the flow through the simulation domain. The same technique used at the inflow boundary can be used for the outflow one. However, a slightly different treatment is adopted at the outlet to reduce the computational time. When an inner‐fluid particle goes across the outflow boundary line, it becomes an outflow particle, and the governing equations are not solved on the particle anymore, but its properties are kept unchanged when it moves through the outflow region. This treatment is similar to that used by Federico *et al.* 22, in which the properties of outflow particles are frozen. Finally, the particles are removed from the computational domain when they pass through the outlet threshold line (Figure 1).

To check whether the inflow/outflow boundary condition satisfies the volume conservation or not, we simply calculated the volume flows inside the computational domain at the inlet as well as that flows out of the domain at the outlet boundary at every second of the simulation for several test cases, and we found out the maximum difference between the inlet and outlet volumes is less than 0.5%. This shows the validity of volume conservation on the inflow/outflow boundary condition in the present simulations. However, for a detailed modelling of inflow/outflow boundary conditions, we need to refer to Hosseini and Feng 48 where a rotational pressure‐correction scheme with consistent pressure boundary condition is proposed to overcome the numerical difficulties and consistently implement the inflow/outflow boundary conditions.

#### 2.3.2 Treatment of rough bed boundary

Because a rough bed with relatively large roughness elements is studied in the present work, an important question arises regarding where exactly the location of the zero‐velocity plane (also called numerical bed level in Figure 1) would be. In the present model, the vertical level of the zero‐velocity plane is located at some distance below the roughness crest, and fluid particles are placed from this level to the water surface. The drag force model is introduced over the distance between the bed level and the roughness crest, that is, the drag‐induced stress term **τ** *_d_*/*ρ* is calculated only for the fluid particles that are located between the numerical bed level and the crest of roughness zone (Figure 1). This distance is named the effective roughness height or the thickness of the roughness zone (*R_d_*) and is assumed to be variable for different flow conditions as according to experimental observations, the effect of bed roughness on the flow differs for different flow conditions. The numerical bed elevation that defines the base of the roughness zone can be considered as the zero‐velocity plane on which the spatial and temporally averaged flow velocity drops to 0. For this bed boundary, several layers of dummy particles (red particles in Figure 1) are placed below the boundary line to address the truncated kernel area in the vicinity of the boundary. The velocity of these dummy particles is not evolved in the calculations, that is, they are fixed in space with zero velocity, but they have pressure to prevent the fluid particles from penetrating this boundary. In this sense, the zero‐velocity bed level also corresponds to the location of the upper line of dummy particles. In the present WCSPH simulations, the pressures of dummy particles are determined through the equation of state (Eq. (18)) after their density variations have been computed by using the SPH continuity equation (16). This algorithm can ensure that adequate pressure is obtained on the dummy particles to prevent the inner fluid particles penetrating the wall boundary.

**Figure 3:**
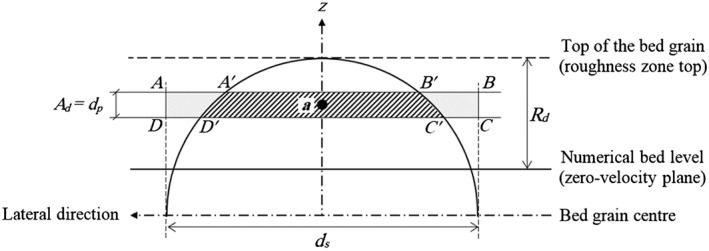
A schematic view of the bed drag force model.

A schematic view of the bed drag force model including the roughness spheres is shown in Figure 3, in which the roughness zone is from the numerical bed level (zero‐velocity plane) to the crest of the sphere with a thickness of *R_d_*. Considering a section normal to the flow direction as depicted in Figure 3, it is assumed that when a fluid particle *a* is located within the roughness zone, the roughness element (the sphere) produces a drag‐induced shear on this particle. This actually exerts a force on the fluid fragment of width *d_s_* and height *d_p_* (*ABCD* in Figure 3), where *d_s_* is the diameter of the roughness sphere and *d_p_* is the computational particle size. Therefore, the cross‐sectional area *A_d_* in Eq. (12) is assumed to be equal to the particle size *d_p_*, and the bed‐parallel planar area *A_τ_* in Eq. (11) is equal to *d_s_d_p_*. Meanwhile, for each fluid particle located in the roughness zone, as depicted in Figure 3, a shape function *W_d_* is defined as the area of part of the water fragment located within the sphere (*A* ^′^ *B* ^′^ *C* ^′^ *D* ^′^ in Figure 3) over the total area of the fragment (*ABCD* = *d_s_d_p_*) by the following equation:

(20) $$W_{d}=\frac{A_{A^{\prime }B^{\prime }C^{\prime }D^{\prime }}}{A_{\text{ABCD}}}\text{.}$$

This function accounts for the shape of the roughness elements that are defined as spheres in the present study to match the roughness elements used in the laboratory study.

Another parameter of Eq. (12) that needs to be considered is the drag coefficient *C_d_*. According to the original work on particle modelling of porous flows using the MPS method by Gotoh and Sakai 31, *C_d_* usually lies between 1.0 and 1.5, and thus, a value of 1.0 is simply adopted in the present study. Different values of *C_d_* have also been described in the literature for spherical particles. In the experiments of Schmeeckle *et al.* 49 on turbulent open channel flow over fixed spheres, the drag coefficient was found to be 0.76. They also measured the drag force in turbulent flows over cubes and natural particles and found that the drag coefficient was significantly higher than that used to model the bed load motion. In the proposed drag force model (Eq. 12), the product of *C_d_W_d_* acts as the total drag coefficient. By assuming half of the bed grain to be the effective roughness height and *C_d_* = 1.0, the average value of *C_d_W_d_* for the particles inside the roughness zone would be equal to 0.785, which is close to the value found by Schmeeckle *et al.* 49 for spherical particles. Here, it should be noted that the roughness spheres as shown in Figure 3 do not physically exist in the numerical model so particles can penetrate inside the roughness zone but feel its influence.

## 3 Model Applications and Results Analysis

### 3.1 Model setup and computational parameters

In this section, an SPH model is developed for uniform turbulent open channel flows over a sloping rough bed and validated by the depth and velocity data obtained from Particle Image Velocimetry (PIV) measurement in a laboratory channel with uniform‐sized spheres packed in a hexagonal pattern on the bed 50. In these tests, the bed sphere diameter *d_s_* is 24 mm, and the channel slope *S*_0_ ranges from 0.002 to 0.004. For this application, a rectangular computational domain is chosen with a length of *L* = 4*H*, where *H* is the water depth. Three layers of fixed dummy particles are used for the bottom wall, and three layers of moving particles are used for the inflow as well as outflow regions to satisfy the complete kernel area of the inner‐fluid particles near the boundary lines (Figure 1). Because the effect of bottom roughness on the flow depends not only on the absolute roughness size but also on the flow conditions, 12 test cases with different bed slopes and water depths are simulated to assess the accuracy of the drag force model in addressing the roughness effect. Relevant parameters used in the test cases are summarized in Table 1. According to this table, the *Fr* number for all 12 cases is below 1, which means all tests are in the sub‐critical flow condition, while Chang and Chang 34 and Chang *et al.* 35 covered more flow regimes. Meanwhile, the domain is discretized by SPH particles with size *d_p_* = 2 mm to have at least 20 particles over the depth for the shallowest case (*H* = 40 mm). The smoothing length is taken to be 1.2*d_p_* in the present study. This value has been recommended as the most appropriate SPH smoothing length in many studies as common practice. Because the interfacial boundary layer in the physical model between the bed roughness and the free flow is expected to be quite thin, a kernel function with a narrower influence domain but steeper slope near the central point is expected to be more adequate. Therefore, the cubic spline function of Monaghan and Lattanzio 51 is chosen for the present simulations. However, an in‐depth investigation is required for the choice of spatial resolution, smoothing length and kernel function in cases where the flow velocity changes sharply over an interfacial boundary layer as in the present study.

**Table 1:** Computational parameters used in test cases (the first four letters and numbers in the test ID show the bed slope, and the rest show the water depth).

Test no.	Test ID	*S*_0_	*H* (mm)	*u* ^*^ (m/s)	*Re*	*Fr*	Calibration/validation
1	S004H40	0.004	40	0.0396	10 843	0.433	C
2	S004H50	0.004	50	0.0443	15 067	0.430	V
3	S004H70	0.004	70	0.0524	32 703	0.564	V
4	S004H90	0.004	90	0.0594	47 301	0.559	V
5	S004H100	0.004	100	0.0626	59 698	0.603	C
6	S003H50	0.003	50	0.0384	11 615	0.332	C
7	S003H60	0.003	60	0.0420	19 516	0.424	V
8	S003H70	0.003	70	0.0454	27 926	0.481	C
9	S003H80	0.003	80	0.0485	32 089	0.453	V
10	S002H60	0.002	60	0.0343	12 022	0.261	C
11	S002H70	0.002	70	0.0371	19 671	0.339	V
12	S002H80	0.002	80	0.0396	30 794	0.435	C

As illustrated in Table 1, the model has been applied to different flow conditions with bed slopes 0.002, 0.003 and 0.004, and water depths from 40 to 100 mm. As mentioned in the previous section, the thickness of the roughness zone (*R_d_*) is assumed to vary depending on the flow conditions. Therefore, six of the test cases (nos. 1, 5, 6, 8, 10 and 12) are used to calibrate the model in terms of *R_d_* by numerical trials when the computed mean velocity profiles achieved the best fit with the experimental data and then a semi‐empirical fitting function is obtained to establish the relationship between the flow depth and relative roughness height *R_d_*/*H*. Based on this, the additional test cases (nos. 2, 3, 4, 7, 9 and 11) are used to validate the model. The calibration tests are selected to cover most of the depth range from 40 to 100 mm and at least two cases of each bed slope. Calibration and validation tests are indicated by letters C and V, respectively, in Table 1.

**Figure 4:**
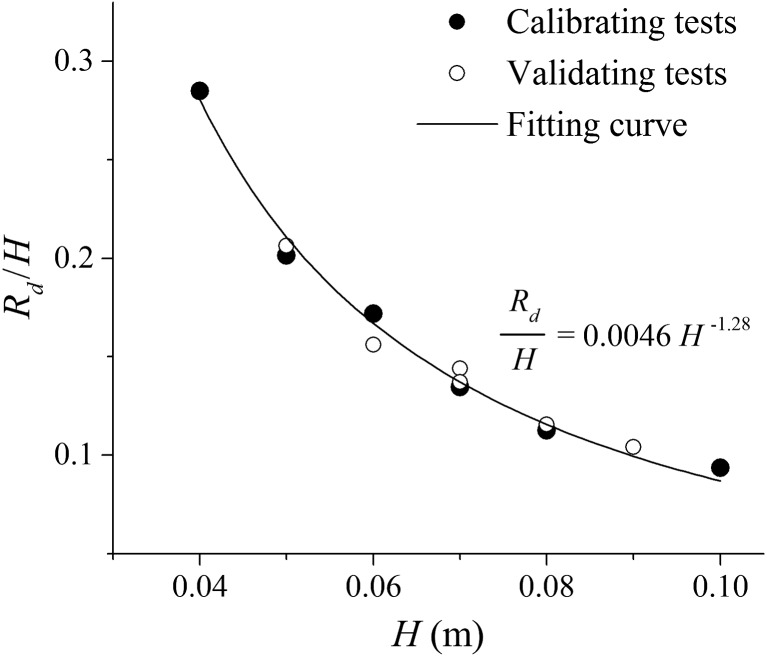
Calibration and validation of the model in terms of the effective roughness height versus the water depth.

The calibration process is as follows. Each test case is simulated by using several *R_d_* values, and the mean absolute error (MAE) between the numerical and experimental velocity profiles is calculated for each one; then, the *R_d_* value corresponding to the minimum MAE is selected as the thickness of the roughness zone for that test case. After running the model for calibrating tests and finding the best *R_d_* with the smallest MAE, the relative roughness height *R_d_*/*H* is plotted against depth *H* (Figure 4), and a curve is fitted to the points using a power function as shown in the figure. For each validating case, different values of *R_d_* are examined in the simulations, and the one with the minimum MAE is used for the test case and plotted on the same graph to see if it follows the fitted curve. As can be seen, the *R_d_*/*H* values of the validation tests have nearly the same relation with the water depth. Further evidence of the model validations will be demonstrated by the good agreement between the numerical and experimental velocity and shear stress distributions along the flow depth, as detailed in the next section.

### 3.2 Analysis of velocity profiles

**Figure 5:**
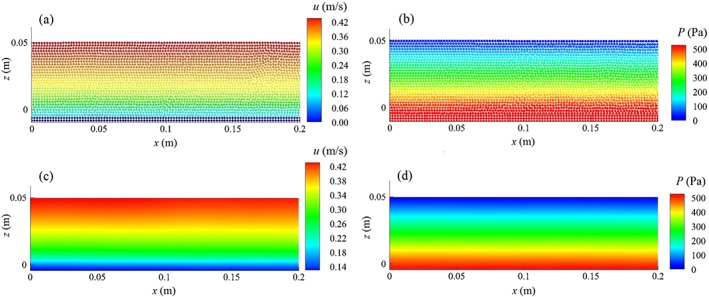
Uniform flow condition (test case S004H50): (a) instantaneous streamwise velocity; (b) instantaneous pressure; (c) time‐averaged streamwise velocity; and (d) time‐averaged pressure.

**Figure 6:**
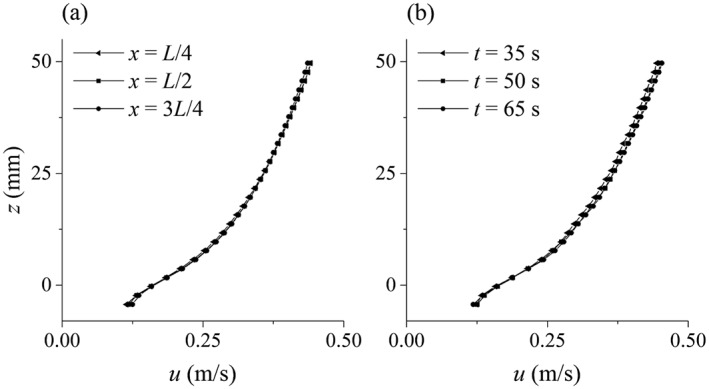
Uniformity and steadiness of the flow (test case S004H50): (a) time‐averaged velocity in three sections through the channel and (b) space‐averaged velocity in three times with 15‐s intervals.

Figure 5(a) and (b) presents the instantaneous streamwise velocity and pressure (*t* = 70 s), and Figure 5(c) and (d) shows their time‐averaged contours, respectively, for the case S004H50. The averaging has been performed over 20 s from *t* = 70 to 90 s. It shows that the uniform flow condition has been successfully imposed by the proposed inflow/outflow boundary technique. This is also shown in Figure 6(a) where the time‐averaged velocity of three different sections of the channel (*x* = 0.25 *L*, 0.50 *L* and 0.75 *L*) are plotted. It is found that the depth‐averaged velocity at these three sections has a maximum difference of 0.5%. Figure 6(b) shows the space‐averaged velocity at three different times (*t* = 35, 50 and 65 s). The maximum difference of the depth‐averaged velocity between these times is 1.96%. This small change in the velocity profile over time also shows the steadiness of the flow. In the present computations, the time to reach the steady state is not exactly the same for all test cases. However, to determine a threshold, it is confirmed that it takes around 20–30 s to achieve the steady flow condition for all 12 cases. The criterion used to define if the flow reaches the steady state is that if the differences of the depth‐averaged value of the space‐averaged (but not instantaneous) velocities at the mid‐section of the channel at different times become less than 2.0%, then the flow is regarded as being steady. The bed drag‐induced shear term removes a part of the flow momentum, and this effect is transferred to the upper layers of the flow by the turbulence model. As a result, the unbalanced momentum transfer occurs during the first 20–30 s, and then the flow gradually reaches the steady state and all time‐averaged flow parameters, for example, velocity and shear stress remain unchanged. In the inflow and outflow boundaries, the flow characteristics are assumed to be unknown rather than being given prescribed values of the pressure and velocity. Therefore, the proposed SPH inflow boundary model is more general in that it does not need experimentally measured or analytically prescribed flow data at the inflow boundary and can thus be applied to more complex flow situations. In Federico *et al.* 22, the model verification was based on the fact that the initial inner velocity field, which was initialized with the analytical solutions and updated by the upstream inflow boundary condition (which was also initialized by the analytical solutions), could be stably maintained or not during the computations. In comparison, in the present SPH inflow model, the open channel flows are generated naturally by following the channel conditions.

**Figure 7:**
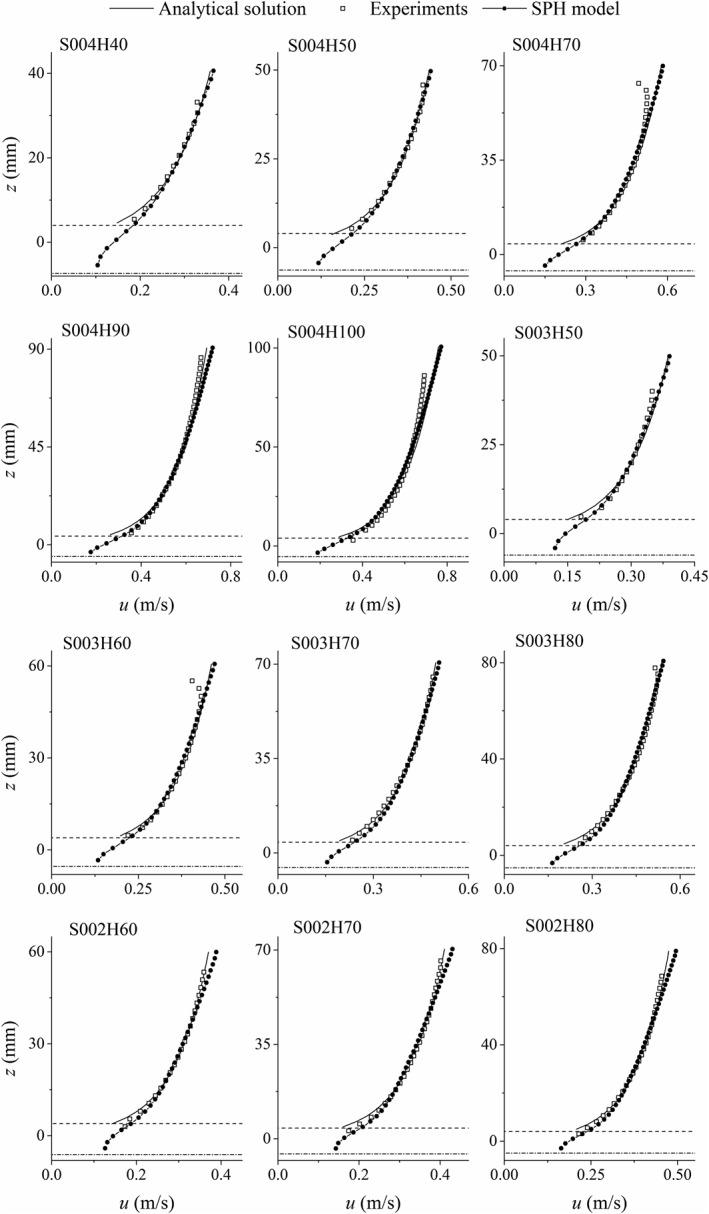
Distribution of the time‐averaged streamwise velocity over depth. Dash‐dotted and dashed lines show the level of the numerical bed (zero‐velocity plane) and the crest of the roughness zone, respectively.

The numerical results of time‐averaged streamwise velocity profiles obtained by using the best‐fit values of *R_d_* are presented in Figure 7, in comparison with the experimental data as well as the analytical profiles that are obtained from the log law. These include all the test cases as indicated in Table 1. The analytical velocity profile is presented in Eq. (21) where *z* is vertical coordinate, *k_s_* is the Nikuradse roughness size and *B_r_* is the logarithmic integration constant that is equal to 8.5 for rough bed uniform flow. We know that as the depth is very shallow and the bed is fully rough, the log law may not be valid. Here, the analytical profiles are used to compare with the numerical results and investigate if the model is able to predict the logarithmic velocity distribution above the roughness zone. The values of *R_d_* as well as MAE of velocity profiles of all test cases are presented in Table 2. Both Figure 7 and Table 2 demonstrate the satisfactory performance of the SPH modelling technique in these proposed flow conditions.

(21) $$\frac{u}{u_{*}}=\frac{1}{k}\ln\left(\frac{z}{k_{s}}\right)+B_{r}\text{.}$$

**Table 2:** Relative roughness heights and numerical errors of all tests.

Test no.	Test ID	*R_d_*/*H*	MAE of *u* (m/s)	MAE of ∂*u*/∂*z* (1/s)
1	S004H40	0.285	0.0052	0.77
2	S004H50	0.206	0.0060	1.17
3	S004H70	0.144	0.0100	1.27
4	S004H90	0.104	0.0100	0.77
5	S004H100	0.094	0.0179	1.25
6	S003H50	0.202	0.0047	1.40
7	S003H60	0.156	0.0063	1.39
8	S003H70	0.135	0.0078	0.67
9	S003H80	0.116	0.0080	1.11
10	S002H60	0.172	0.0052	1.05
11	S002H70	0.137	0.0061	0.81
12	S002H80	0.113	0.0061	0.82

MAE, mean absolute error.

**Figure 8:**
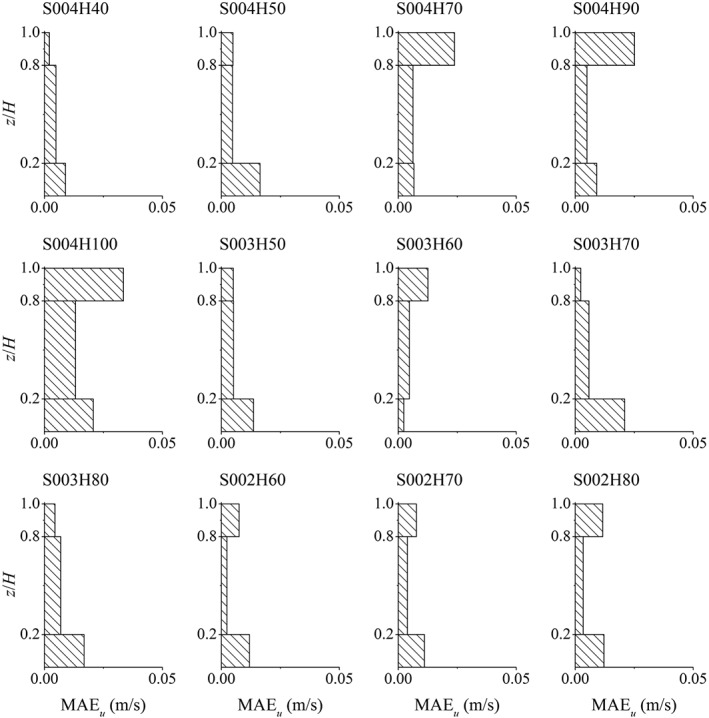
Mean absolute error (MAE) of the streamwise velocity in the lower 20%, middle 60% and upper 20% of the depth.

**Figure 9:**
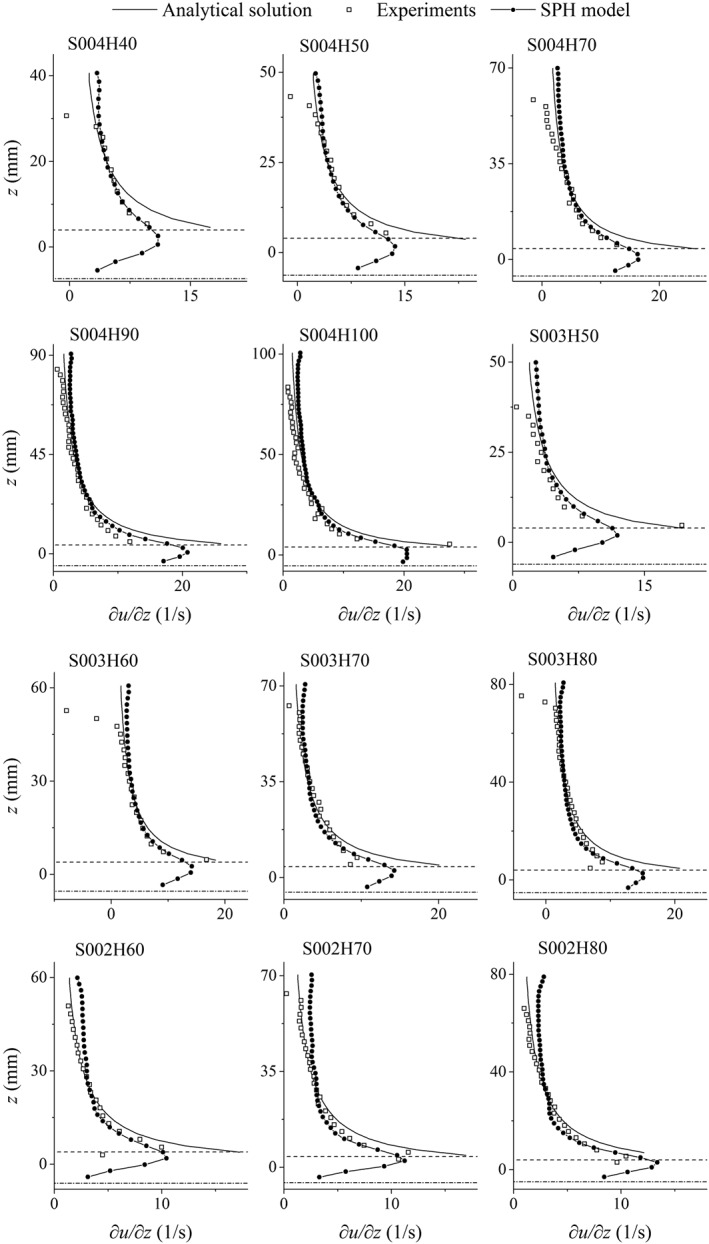
Distribution of the gradient of the time‐averaged streamwise velocity over depth. Dash‐dotted and dashed lines show the level of the numerical bed (zero‐velocity plane) and the crest of the roughness zone, respectively.

**Figure 10:**
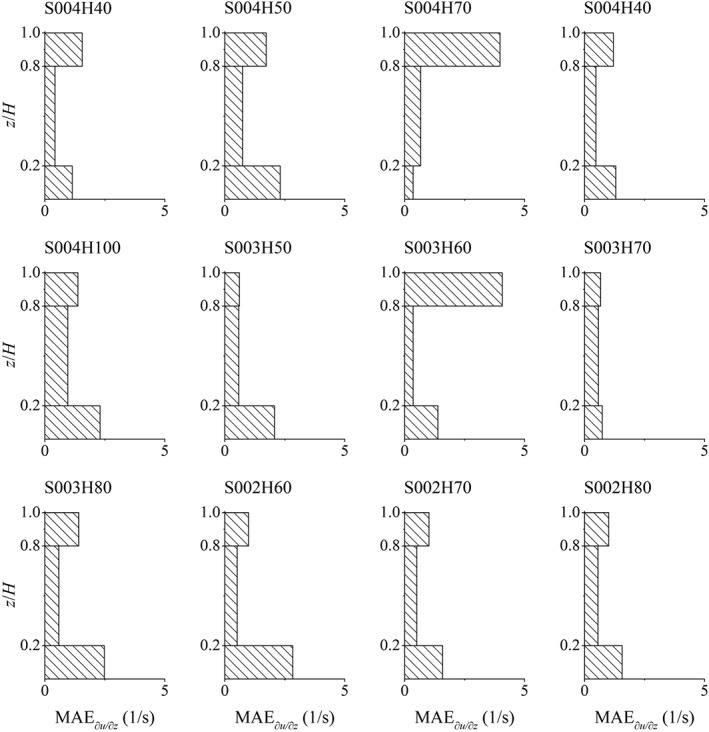
Mean absolute error (MAE) of the streamwise velocity gradient in the lower 20%, middle 60% and upper 20% of the depth.

To determine the error distribution over depth, the MAE is calculated separately in three parts of the depth for each test case, that is, lower 20%, middle 60% and upper 20% of the depth. The purpose of this is to investigate the hypothesis 50 that the bottom 20% of the water depth would be the logarithmic layer and then the upper layers of the flow could be split up differently. This is shown in Figure 8. As the slope of the velocity profile (∂*u*/∂*z*) is also of interest, its distribution is presented in Figure 9 for all test cases, and the values of MAE of these profiles are also calculated. The MAE values of ∂*u*/∂*z* are presented in Table 2, and their distributions in the lower 20%, middle 60% and upper 20% of the depth for all cases are illustrated in Figure 10.

According to Figure 8, with increasing depth, the velocity MAE of the upper 20% of the depth mostly increases, and as the slope decreases, the MAE of the near‐bed velocity generally increases. In most test cases, the lowest MAE of the velocity profiles takes place in the middle part of the depth. This is also valid for the MAE of the velocity gradient profiles as can be seen in Figure 10. Compared with the velocity, the errors of the velocity gradient are usually larger in the lower 20% of the depth. However, in some cases (e.g. S004H70 and S003H60), there seems to be a large error in the upper part of the depth due to the fact that the predicted and measured gradients have different signs near the water surface. Just below the surface, the experimental velocity gradient declines sharply to 0 or even to negative values in some cases, while a non‐zero, but small positive velocity gradient is predicted by the numerical model (Figure 9). The negative gradient in the top of the flow could be due to the fact that the data are derived from a 3D experimental model in which secondary flow circulations occur, while such circulations are not accounted for in the present 2D numerical model. However, the log law (Eq. (21)) presents a positive small, but non‐zero velocity gradient at the top (Figure 9), which is much more similar to the numerical profiles than the experimental ones. This is because the mixing‐length model (Eq. (9)) adopted by the SPH approach has been based on the log law theory. In the mixing‐length formula of Nezu and Rodi 41, it is assumed that above a certain elevation, the mixing length decreases to 0 at the water surface as the size of turbulent eddies is significantly restricted by the surface. Assuming such a decline in the mixing length could lead to a non‐zero velocity gradient near the water surface. On the other hand, the differences in the near‐bed velocity gradient between the numerical and experimental profiles are much less than those between the analytical and experimental ones. This is attributed to the adoption of the robust drag force model by which the near‐bed velocity is related to the shear from the roughness elements rather than assuming a logarithmic distribution in the shear boundary.

### 3.3 Analysis of roughness height

**Figure 11:**
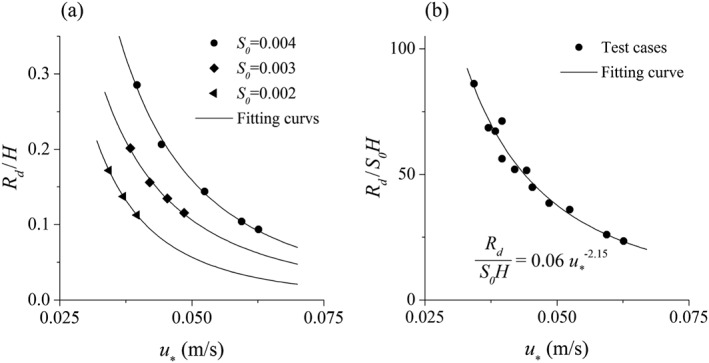
Relative roughness height against shear velocity: (a) relationship between *R_d_*/*H* and u_*_ for different bed slopes and (b) relationship between *R_d_*/*S*_0_ *H* and u_*_ for all tests.

During the calibration/validation process (Section 3.1), the *R_d_* values corresponding to the minimum errors, if divided by the water depth, showed a relationship with the depth based on the power function as presented in Figure 4. According to this figure, as the depth increases, the relative roughness height (*R_d_*/*H*) decreases. It is notable that the bed roughness sphere size is fixed in the present study (*d_s_* = 24 mm). Therefore, *R_d_*/*H* decreases with a decrease in the ratio of roughness size to water depth (*d_s_*/*H*) and vice versa. In this work, the bed roughness configuration is kept constant to study its effect under different flow conditions. As the depth is not the only parameter affecting the flow condition and the bed slope is also involved, we also explored the relationship between the relative roughness height (*R_d_*/*H*) and the shear velocity u_*_. The result is shown in Figure 11(a) with different power fitting functions for different bed slope values. It is shown that the fitting curves are nearly equally spaced with a vertical shift upwards as the bed slope increases and the SPH computational points fall close to the appropriate curves.

It can also be seen that an increase in the shear velocity causes the numerical relative roughness height to become milder for all bed slopes. To provide a single relationship between the relative roughness height and the flow condition, *R_d_*/*S*_0_ *H* of all cases are plotted against u_*_ in Figure 11(b) with the same type of power fitting curve. This also shows that as the flow becomes more sheared (larger u_*_), smaller relative roughness height is required to simulate the experimental condition. In other words, as the ratio of bed roughness to water depth (*d_s_*/*H*) becomes smaller, that is, when the flow depth becomes deeper, a weaker bed effect is generated by the proposed drag force model. However, the magnitude of the form‐drag shear term could be larger for the cases with higher u_*_ because the near‐bed flow velocity is faster.

### 3.4 Analysis of form‐drag and turbulent shear stress

**Figure 12:**
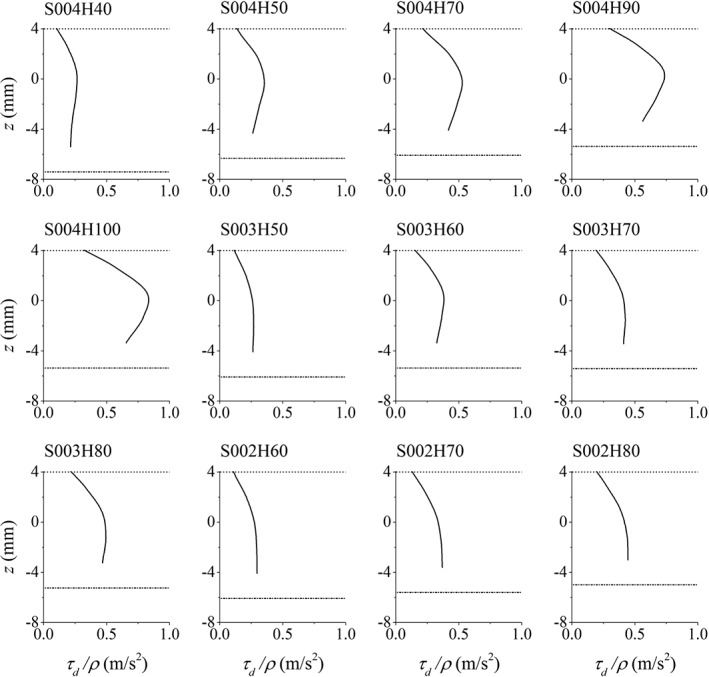
Distribution of the drag‐induced shear term in the effective roughness zone (solid line). Dash‐dotted and dotted lines show the level of the numerical bed (zero‐velocity plane) and the crest of the roughness zone, respectively.

**Figure 13:**
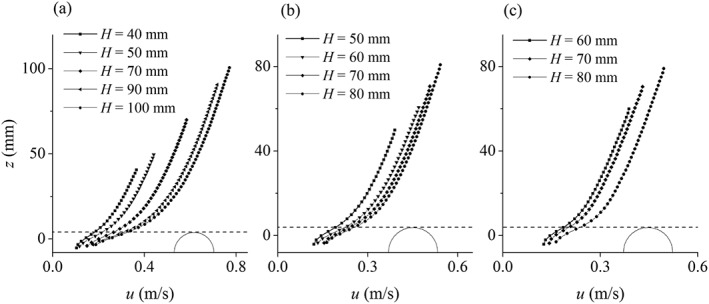
Velocity profiles of tests with bed slopes (a) 0.004, (b) 0.003 and (c) 0.002. The dashed lines show the level of the roughness crest, and the solid half circles schematically depict the roughness element.

The distribution of the streamwise form‐drag shear term (*τ_d_*/*ρ*) in the effective roughness zone is presented in Figure 12 for all the tests. As expected, the average *τ_d_*/*ρ* is larger for cases with higher u_*_ or *Re* number. In other words, where the flow depth is deeper and/or the bed slope is steeper, the form‐drag shear term is larger because of the higher velocity. In most tests, the streamwise *τ_d_*/*ρ* increases with depth to some distance above the wall (zero‐velocity plane) and then decreases to the crest of the roughness zone although the velocity increases in this zone. This decrease can be the result of the shape function in Eq. (20) that declines sharply below the roughness crest. The shape function leads to a non‐constant drag coefficient in the roughness zone that is related to the shape of the elements. In the present simulations, the dominant velocity is the streamwise one, and the contribution of the vertical velocity to the form drag is very small so that it is reasonable to be neglected. It has been found that in the roughness zone, the scale of the time‐averaged vertical velocity is less than 0.5% of the time‐averaged streamwise velocity in our test cases, while it is about 1.0% to 2.0% in the presented 3D experimental data. The underestimation of the vertical velocity in the roughness zone could be due to that the physical dispersion in the vertical direction that is from the obstruction of the flow by the bed elements has not been numerically defined, because the governing equations and the computational domain are discretized at a macroscopic scale. In Figure 13, the numerical results of the streamwise velocity profiles of tests of bed slope 0.004, 0.003 and 0.002 are plotted in separate graphs in order to illustrate the effect of rough bed boundary on the streamwise flow velocity. As can be seen for each bed slope, the velocity is higher for larger depths, and this effect is simulated by variable roughness height in the model.

**Figure 14:**
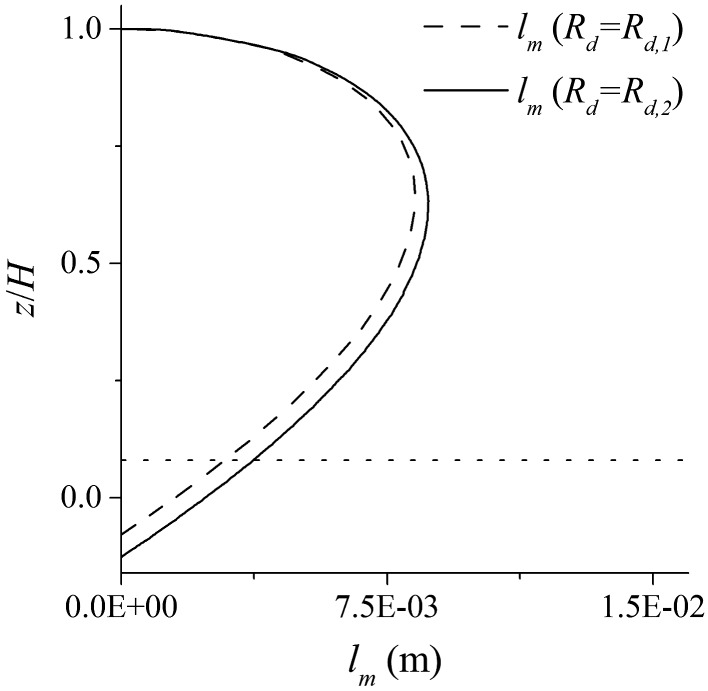
Distribution of the mixing length in two cases with the same depth (*H* = 50 mm) and different effective roughness heights (*R*_*d*,2_ > *R*_*d*,1_). The zero reference of the mixing length is on the numerical bed level (zero‐velocity plane), and the dotted line shows the crest of the roughness zone.

Using a variable *R_d_* in the model affects not only the drag shear term but also the turbulent shear stress near the bed. In the present model, the zero reference datum for the mixing length is defined by the zero‐velocity plane of the flow. This is illustrated in Figure 14 where *l_m_* is plotted for two cases with effective roughness heights of *R*_*d*,*1*_ and *R*_*d*,*2*_ (*R*_*d*,*2*_ > *R*_*d*,*1*_). Here, the eddy‐viscosity is higher when the thickness of the roughness zone (*R_d_*) is larger; thus, the shear stress calculated by Eq. (4) is also larger. This leads to a higher impact of the bottom drag effect on the upper flow. In general, any changes of *R_d_* could affect both the drag force and the turbulence models, and thus, the simulated flows will change. It is also notable that a small change in the mixing length, on the crest of the roughness zone, could have a considerable effect on the eddy‐viscosity (Eq. (8)) because the velocity gradient (or the local strain rate |**S**|) is at a maximum on this interface.

**Figure 15:**
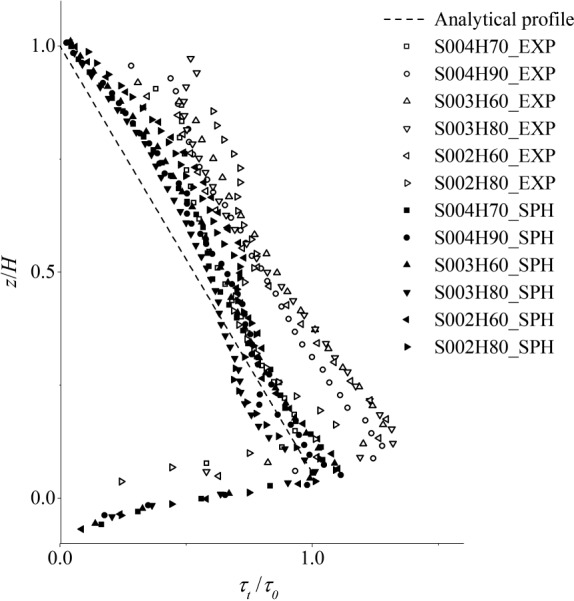
Distributions of the normalized turbulent shear stress with depth.

For six of the 12 test cases shown in Table 1, the profiles of the time‐averaged shear stress estimated by the SPS with the mixing‐length model are presented in Figure 15 in comparison with the experimental data and with the analytical profile obtained from Eq. (22). In this equation, *τ*_0_ is the shear stress at the bed that is estimated by *ρgHS*_0_.

(22) $$\tau =\tau_{0}\left(1-\frac{z}{H}\right)\text{.}$$

To better illustrate the data, the horizontal axis is normalized by *τ*_0_, and the vertical one is normalized by the flow depth *H*. As can be seen, the numerical computations underestimate the experimental shear stresses, although they are in a fairly good agreement with the analytical solution. It is notable that the experimental data are taken from a 3D flow over a rough bed surface, which could lead to that they exceed the analytical shear stresses by about 20–30%. Besides, the underestimation of the experimental shear stress by the numerical model is also related to the dimensional differences, as in the present 2D model, the shear stress in the lateral direction is neglected. The width‐wise shear stress is the result of steady streaming in the form of flow circulations in the lateral direction. In spite of this, the 2D SPS model is still able to give satisfactory results in the uniform flow because the effect of the lateral shear stress is very much smaller compared with the streamwise one. Moreover, the close collapses of six SPH data along almost a single line indicate the consistency and convergence of the numerical simulations.

**Figure 16:**
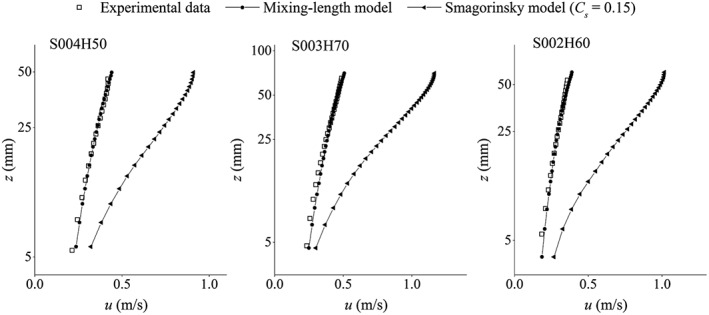
Time‐averaged streamwise velocity obtained from the present mixing‐length model compared with the one obtained from the Smagorinsky model with *Cs* = 0.15 and the experimental data for test cases S004H50, S003H70 and S002H60 (vertical axis *z* is in logarithmic scale).

**Figure 17:**
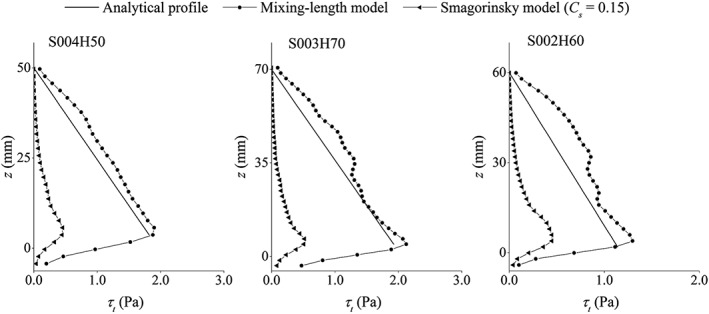
The *x‐z* component of the turbulent shear stress obtained from the present mixing‐length model compared with the one obtained from the Smagorinsky model with *Cs* = 0.15 and the analytical profiles for test cases S004H50, S003H70 and S002H60.

As mentioned before, the eddy‐viscosity model with a Smagorinsky constant in the range of 0.1–0.15 is not able to estimate the correct amount of turbulent shear stress in a uniform open channel flow over a rough bed boundary. To investigate this issue, here, we repeat the simulations of three test cases S004H50, S003H70 and S002H60 by using the Smagorinsky model with *C_s_* = 0.15. The result is presented in Figures 16 and 17, where, respectively, the streamwise velocity and shear stress profiles are compared with the ones obtained from the present mixing‐length eddy‐viscosity model. Meanwhile, the experimental velocity profiles and the analytical shear stress profiles are also presented for a comparison. As can be seen, the shear stress is consistently largely underestimated, leading to the overestimation of the velocity.

In contrast to their DNS results with good agreement with the reference data, Mayrhofer *et al.* 37 observed the overestimation of the velocity in their SPH‐LES computations of a wall‐bounded channel flow with friction *Re* of 1000, where an eddy‐viscosity model was used with a Smagorinsky constant *C_s_* = 0.065. They pointed out that the correct representation of energy redistribution between Reynolds stress components in an SPH‐LES framework would require 16 times finer resolution than needed in a classic Eulerian LES one. They stated that the most obvious solution is an increase in the resolution but it also highly increases the computational cost. Finally, they concluded that the underperformance of their LES was due to the problems inherent in the standard SPH discretizations related to the pressure–velocity interactions.

In the present study, the friction *Re* is even higher than that in the study of Mayrhofer *et al.* 37, and on the other hand, the resolution is also quite coarse. Therefore, the insufficiency of the LES with the standard Smagorinsky model becomes more obvious in the present simulations. In addition, the rough bed boundary is another important influence factor too. When filtering the discretized equations using an SPH kernel function to represent the turbulence effect, a part of the turbulent stress that is mainly due to the spatial filtering has been lost by the standard eddy‐viscosity model (with Smagorinsky constant). This issue becomes even more important when the discretized flow velocity changes sharply over the filtering volume/area, for example, at the interfacial boundary between the roughness zone and the free flow in the present study. Besides, the rough bed boundary has a dominant effect on the whole water depth, so non‐accurate parameterization of the turbulence effect at this boundary makes significant errors in the whole flow domain. However, if the eddy‐viscosity model is adequately parameterized, reasonable results can still be obtained. As a result, we have applied the mixing‐length model of Nezu and Rodi 41, which is on the basis of physical measurements, in order to recover that part of the turbulent stress that cannot be captured by the standard Smagorinsky model with small *C_s_*.

Nonetheless, one shortcoming of the proposed turbulence model is that the eddy‐viscosity coefficient is physically defined so it is not dependent on the computational resolution. In other words, if one uses a smaller particle size (higher resolution), the resolved part of the turbulence stress would become higher, but the mixing‐length product that is the representative of the unresolved part would not decrease with the discretization and/or filtering size. Thus, the total turbulent stresses could be overestimated in the cases with higher resolution. Accordingly, the flow velocities would be expected to be underestimated in such a situation. It is promising to note that the present mixing‐length approach works quite effectively with the SPH when relatively coarse particle resolution is used, which is the case in most practical engineering applications.

## 4 Conclusion

In this study, an SPH model has been developed to simulate the turbulent open channel flows over rough bed boundaries based on the solution of 2D N‐S equations including two additional stress terms to account for the flow turbulence and bed roughness effect. As shown, the standard Smagorinsky‐based SPS model with a fixed *C_s_* = 0.15 was unable to reproduce the correct shear mechanisms in uniform open channel flows. Therefore, a mixing‐length model has been applied to calculate the turbulent eddy‐viscosity. A drag force model has been developed to account for the bed roughness effect, in which a shape function is introduced to consider the geometry of the bed surface roughness elements. Meanwhile, an efficient inflow/outflow boundary treatment has been proposed and demonstrated to generate a stable flow simulation without the need to use prescribed velocities at the flow inlet.

Twelve test cases of different bed slopes and water depths have been simulated to investigate the effect of bed roughness under various flow conditions. A roughness zone is defined near the rough bed boundary where a form‐induced drag shear term is applied on the SPH particles. The thickness of this zone (*R_d_*) is assumed to be flow dependent, such as being related to the flow depth *H* and the shear velocity on bed u_*_. The model results showed good agreement with the experimental data as well as the analytical solutions in view of the velocity and shear stress profiles. This confirms that the bed roughness effect has been successfully addressed by the drag force model and the transport of this effect to the upper layers of the flow has been correctly reproduced by the proposed turbulent mixing‐length approach. Because the governing equations, as well as the computational domain, are discretized at a macroscopic scale in the roughness zone, the physical dispersion in the vertical direction is disregarded. Thus, the flow shear is dominantly driven in the streamwise direction but transported vertically by the turbulence closure. The computed streamwise velocity and shear stress profiles suggested that this assumption has not caused substantial errors for the 12 flow test cases and the macro flow behaviours have been well reproduced. This is due to the turbulence model correctly modelling the shear transfer from the roughness layer to the free flow.

Whether the inaccuracy of the SPH‐LES approach in wall‐bounded channel flows is related to the pressure–velocity interactions (as addressed by 37) or to the deficiency of the standard Smagorinsky model, the proposed mixing‐length approach is able to overcome this difficulty due to the eddy‐viscosity being realistically parameterized. However, as the mixing length is independent of the computational resolution, it may overestimate the shear stress in cases with higher particle resolution that may cause an underestimation of the flow velocity. Therefore, this model is proposed to be coupled with the SPH when coarse discretization of the equations is considered, unless an effective method is found to link the mixing length to the spatial discretization so as to enhance the capacity of the model. In addition to this, the method of filtering the governing equations with different kernel functions needs to be investigated in more detail because of the existence of the rough bed boundary over which the flow velocity has a sharp change. These issues along with the effect of various configurations of bed roughness on the flow resistance are considered as future studies.
